# CMR in pediatric patients with congenital heart disease: comparison at 1.5T and at 3.0T

**DOI:** 10.1186/1532-429X-14-S1-P119

**Published:** 2012-02-01

**Authors:** Kim-Lien Nguyen, Sarah Khan, John Moriarty, Kiyarash Mohajer, Pierangelo Renella, Gary Satou, Ihab Ayad, Swati Patel, Ines Boechat, J Paul Finn

**Affiliations:** 1 Division of Cardiology, David Geffen School of Medicine at UCLA, Los Angeles, CA, USA; 2 Laboratory of Cardiac Energetics, National Heart, Lung, and Blood Institute, Bethesda, MD, USA; 3 Division of Radiological Sciences, David Geffen School of Medicine at UCLA, Los Angeles, CA, USA

## Background

Despite the theoretical advantages of higher field strength, widespread adoption of CMR at 3.0T has been slow. To the best of our knowledge, there have been no published reports on the use of 3.0T for imaging in pediatric congenital heart disease (CHD). We sought to assess the feasibility of CMR in pediatric patients with CHD at 3.0T and to compare the technical and diagnostic performance with an age-matched and clinically comparable control group at 1.5T.

## Methods

Forty-six pediatric patients with suspected or known CHD were referred for clinical CMR evaluation. Twenty-three underwent imaging at 1.5T (age 1 day to 7.8 years, mean 28.7±33 months) and 23 underwent imaging at 3.0T (age 3 days to 8 years, mean 47.8±31.4 months). SSFP cine imaging, time-resolved magnetic resonance angiography (TR-MRA), and high resolution contrast-enhanced MRA (CE-MRA) were performed routinely. Two readers independently evaluated the data sets for overall image quality, thoraco-abdominal vessel and cardiac chamber definition, and presence of artefacts. SNR and CNR from both data sets were calculated.

## Results

95% of SSFP cine images at 3T were rated as good or excellent image quality with 73% having mild and 24% having moderate artefacts (k=0.07). 100% of Arterial and venous phase CE-MRA images were considered good or excellent quality (k=0.18, k=0.23 respectively). Cardiac chamber definition was considered good or excellent in 95% of arterial and venous phase CE-MRA images (k=0.08). 100% of Arterial and venous phase CE-MRA images showed good or excellent definition of the thoraco-abdominal vessels (k=0.08). For SSFP cine images, the SNR were 45.0±22.3 and 19.0±6.3 (P<0.01) at 3T and 1.5T respectively. The CNR was 25.7±20.0 vs 7.8±5.2 (P<0.01). For CE-MRA images, SNR was 31.7±10.9 vs 24.3±11.9 (P<0.01). CNR was 25.2±10.4 vs 18.4±9.8. TR-MRA maximum enhancement factor was 2.3±1.9 at 3.0T vs 1.7±0.8 at 1.5T (P<0.01). On average, both readers scored cine SSFP images higher at 1.5T and CEMRA images higher at 3.0T. However, overall diagnostic performance was high at both field strengths.

## Conclusions

CMR imaging of paediatric patients with CHD and vascular abnormalities at 3.0T is feasible. Relative to 1.5T, SNR and CNR are both improved at higher field strength and higher resolution CEMRA is achievable. Whereas SSFP artefacts at 3.0T are more prevalent, they rarely render cine imaging non-diagnostic. Both field strengths can be used successfully for cardiac and vascular imaging. The decision as to which to use is weighted by local availability and the relative requirement for detailed vascular vs intra-cardiac imaging.

## Funding

None.

